# Detrimental Changes in Individual Health-Promoting Behaviors Among Internally Displaced Israelis

**DOI:** 10.3389/ijph.2025.1607794

**Published:** 2025-05-21

**Authors:** Naomi Fliss Isakov, Miri Levi-Shahar, Yulia Balmakov, Ranaa Mahajni Yunis, Ronit Endevelt, Moran Blaychfeld Magnazi

**Affiliations:** ^1^ Nutrition Division, Department of Public Health, Ministry of Health, Jerusalem, Israel; ^2^ Department of Health Promotion, School of Public Health, Faculty of Medicine, Tel-Aviv University, Tel Aviv, Israel; ^3^ Department of Education, Efrata Collage of Education, Jerusalem, Israel; ^4^ Faculty of Medicine, Hebrew University of Jerusalem, Jerusalem, Israel; ^5^ School of Public Health, Haifa University, Haifa, Israel

**Keywords:** health promotion, obesity, healthy lifestyle, armed conflicts, displaced population

## Abstract

**Objectives:**

Israel has forcibly displaced more than 200,000 people to hotels or apartments, due to armed conflict. Our study aimed to identify changes in health-promoting behaviors during displacement.

**Methods:**

Two online surveys were conducted assessing health-promoting behaviors and body weight before and during displacement. Univariate and multivariate analyses were performed to compare displaced and non-displaced respondents.

**Results:**

A total of 997 and 153 participants completed the first and second surveys respectively. A significant decrease in diet quality (P < 0.001), physical activity frequency (P = 0.016), and an increase in diet quantity (P < 0.001) and weight (P = 0.001) were reported among displaced individuals, compared to non-displaced individuals. Living with children, and a healthy pre-war lifestyle were found to be independently protective of detrimental lifestyle change (OR = 0.46, 95% CI0.28–0.76 and OR = 0.63, 95% CI0.40–0.97), risk factors (OR = 2.40, 95% CI1.30–4.43 and OR = 3.54, 95% CI1.71–7.32), for detrimental lifestyle changes.

**Conclusion:**

Detrimental changes to health promoting behaviors were reported in all study groups, although they were significantly higher in displaced individuals. Immediate changes did not differ between respondents staying in hotels or apartments, but were sustained only in hotels.

## Introduction

Armed conflicts, economic crises, and natural disasters cause countries to displace their populations to safer places. Forced displacement due to armed conflict is driven by a combination of direct threats to life, destruction of essential infrastructure, and strategic objectives of warring parties. Displaced populations have been found to be at higher risk of various health-related outcomes, including infectious diseases [1], poor management of chronic illnesses [2–4], mental health issues [5, 6], sleep problems [7–9], food insecurity [10, 11], disability and premature death [1, 4]. In addition, displacement is known to disrupt social stability, and economic security. Populations that have been displaced and resettled have also been found to change their health-seeking behaviors [12]. Recent studies among displaced Ukrainian populations have identified several displacement–related risk factors such as changes in breastfeeding [13], healthy eating [14], and an overall decrease in the attitude and priority of health seeking behaviors [15].

On 7 October 2023, Israel was led to months of immense armed conflict on its southern and northern borders. Immediately after the war broke out, with the invasion of Hamas militants through Israel’s southern borders and the threat of the same scenario from Hezbollah militants from its northern borders, Israel forcibly internally displaced more than 200,000 citizens from their homes to safer places. The Israeli government facilitated the evacuation of its citizens from its northern and southern borders and financed their housing in central Israel. People were urged to leave within hours, temporarily housed in hotels and apartments, separated from their familiar surroundings, daily routine, occupation and community. At the time, the circumstances that would end the war and allow these communities to return to their homes and lives were not anticipated, which led to immense insecurity and confusion. In contrast to these populations, other Israeli families living in the center of the country were not forcibly displaced. None the less, it is very likely that all Israelis were deeply influenced by the war, either through personal and/or family members’ survival of the terror attack on 7 October, through active participation in the war, or through the continued rocket attacks on civilian centers, even in central Israel.

These special circumstances led us to believe that the forcibly displaced populations experienced dramatic changes in their dietary habits, physical activity, smoking, alcohol intake, and consequently, body weight. These factors, in turn, may increase their risk of chronic diseases and are therefore a major public health concern for the Israeli health system. Therefore, we aimed to assess the changes in healthy lifestyle characteristics among forcibly displaced Israelis.

## Methods

### Study Design

An online survey was administered twice during the war to two population samples.

### Study Population

The first online survey was administered between 20 November 2023 and 1 January 2024 (which corresponds to the first and third months of the war), describing an immediate response to the war and displacement. The second online survey was administered between 1 May 2024 and 27 June 2024 (which corresponds to the eighth and ninth months of the war), describing later responses to the war and displacement.

### Data Collection

During data collection of both surveys, they were administered through social media platforms. We had the opportunity to engage with displaced individuals and communities through public health clinicians in displacement sites. As part of the routine regulatory authority of the Nutrition Division of the Ministry of Health, public health dieticians and other healthcare workers were allocated to displacement sites throughout Israel to address the pressing dietary and other medical needs of displaced individuals. Special attention was given to subjects with specific dietary needs (celiac disease, food allergies, medical food dependency etc.), who were evacuated from their homes without the opportunity to prepare in advance. This close contact with this special population allowed us to disseminate the survey among social media groups of communities that were displaced throughout Israel, specifically targeting their responses. Community social media groups gave access to those displaced in hotels, and to those displaced in apartments, enabling us to collect data from both locations. Furthermore, the survey was administered in social media groups of communities in the center of Israel that had not been displaced and remained in their homes during the war.

Data collection for both surveys used a convenience sample of those who chose to answer. The surveys were performed using the same methods of distribution and data collection. Since the questionnaire was anonymous, we could not determine if participants answered the questionnaire twice and compare their results over time.

By answering the questionnaire, participants declared that they consented to participate in this study and that their data would be used for the research needs of the Nutrition Division of the Ministry of Health. Due to the anonymous nature of the data collection and its purpose, the ethics committee of the Ministry of Health approved it and waived the requirement for informed consent.

### Study Survey and Covariate Definitions

The survey was constructed to assess participants’ demographic profile, current locations, and perceptions of lifestyle and dietary quality before and during the war. The survey questionnaire, administered in both the first and second surveys is included as Supplementary Material S1.

Participants described their place of stay during the war as either displaced in hotels, displaced in apartments (self- or government-paid, staying with friends or family) or at home (non-displaced).

Pre-war lifestyle characteristics were defined as:

1) Pre-war diet quality was subjectively rated on a scale of 1–10. Accordingly, a healthy pre-war diet was defined as a pre-war diet quality score of ≥5. 2) Frequency of pre-war physical activity (PA) was rated as 0 to 7 times/week. Regular pre-war PA was defined as a reported PA frequency of ≥3 times per week. 3) Pre-war non-smoking was defined as reporting never smoking or former smoking. 4) Pre-war weight was reported as below normal, normal, or above normal, for age and gender. Perceived pre-war normal body weight was reported for each age and gender. The sum of pre-war health-promoting lifestyle characteristics (pre-war healthy diet, pre-war regular PA, pre-war non-smoking and a pre-war normal body weight) was calculated and a pre-war healthy lifestyle was determined if ≥3 characteristics were determined.

Lifestyle changes during the war were reported in terms of 1) diet quality, 2) diet quantity, 3) PA frequency, 4) alcohol consumption, and 5) body weight. For each of these parameters, participants were asked to subjectively rate their current behavior, compared to their pre-war behavior, on a five-point Likert scale as: significant decrease/slight decrease/no change/slight increase/significant increase. Significant changes in these lifestyle characteristics were determined if participants reported 1) a significant decrease in diet quality, 2) a significant increase in diet quality, 3) a significant decrease in PA frequency, 4) a significant increase in alcohol consumption and 5) a significant increase in body weight. Other than that, participants were also asked to report changes in 6) smoking burden as: no change in smoking status/did not smoke before the war but started smoking during the war/smoking but reduced smoking frequency during the war/smoking, but increased smoking frequency during the war. A significant increase in smoking burden during the war was determined if participants reported either starting to smoke, or increasing smoking frequency during the war.

Detrimental lifestyle changes were determined when ≥3 of 6 lifestyle characteristics were reported as significantly changed.

The frequency of food group consumption was assessed at the time of the survey response. Respondents were asked to report the number of servings of food groups consumed per day/week. The food groups assessed were:

Beneficial food groups: 1) vegetables (fresh, baked or cooked), 2) fruits (fresh, dried or preserved, excluding fruit juice), 3) legumes, 4) whole grains (whole wheat bread and cooked whole grains).

Harmful food groups: 5) sweets and desserts (cakes, cookies, chocolate bars, candy, ice cream), 6) savory snacks, 7) savory baked goods, 8) sugar-sweetened beverages (soft drinks, energy drinks, carbonated beverages, iced tea, iced coffee and fruit juices), 9) processed meats, 10) red meat, 11) processed sauces (ketchup, teriyaki sauce, sweet chili sauce etc.), fast food (fries, onion rings, pizza etc.)

Consumption of more than two servings per day of fruit and vegetables, and more than three servings per week of all other food groups was dichotomously categorized.

### Statistical Analysis

Statistical analyses were performed using SPSS software version 29. The normality of the distribution of participants’ ages was tested using the Shapiro-Wilk test. Since it was found to be normally distributed, it was presented as mean ± Standard Deviation (SD). Dichotomous and categorical variables were presented as absolute values and/or percentages. Differences in age between two groups (displaced groups vs. non-displaced groups) were tested using the independent samples t-test. Differences between groups in dichotomous or categorical variables were tested by Chi-Square. The multivariate association between displacement and place of stay (hotel vs. apartment), was determined using a logistic regression analysis, adjusting for age, gender, religion, chronic disease and one another. Variables that differed significantly between study groups in univariate analysis (pre-war lifestyle, and living with young children) were added to the regression model. The level of significance for all analyses was set at P < 0.05.

## Results

### Study Population

The survey was distributed through social media groups in all regions of Israel. The survey was answered by 1,481 people, but complete questionnaires were collected from 997 (67.3%) participants. A comparison between participants who fully completed the questionnaire and those who did not, is presented in Supplementary Table S1.

A total of 562 participants were displaced from their homes and were staying in hotels, and 150 were staying in apartments. The control group of non-displaced participants was composed of 285 respondents who were at home. The majority of the participants were Jewish (94.5%) and women (82.9%), and 54.8% reported living with young children. Nearly 18% of participants reported being diagnosed with a chronic disease that required dietary treatment or maintenance, including: pre-diabetes/diabetes (6.9%), High blood cholesterol or heart disease (1.8%), and hypertension (2.2%). The sociodemographic, clinical and pre-war lifestyle characteristics of the study population are presented in Table 1. Compared to the displaced, the non-displaced participants had higher proportions of non-Jewish, and ultra-orthodox respondents.

**TABLE 1:** Sociodemographic, clinical and pre-war lifestyle characteristics of the study population, and between group caparison, Israel, 2024.

	Total population (N = 997)	Displaced in hotels (n = 562)	Displaced in apartments (n = 150)	Not displaced (n = 285)	P
Age (mean ± sd)	47.5 ± 16.3	48.0 ± 15.4	46.9 ± 18.4	46.9 ± 16.9	0.599
Gender (n,% female respondents)	827, 82.9	455, 81.0	117, 78.0	255, 89.5	0.001^a^ ^,^ ^b^
Jewish (n,%)	941, 94.5	550, 97.9	145, 96.7	246, 86.6	<0.001^a^ ^,^ ^b^
Chronic diseases (n,%)	176, 17.7	104, 18.6	28, 18.7	44, 15.4	0.499
Living with children (under 18 years) (n,%)	541, 54.8	313, 56.1	61, 40.9	167, 60.1	0.001^b^
Religious affiliation (n,%)					
Secular	794, 79.6	498, 88.6	129, 86.0	167, 58.6	<0.001^a^ ^,^ ^b^
Religious	121, 12.1	58, 10.3	14, 9.3	49, 17.2	
Ultra-orthodox	82, 8.2	6, 1.1	7, 4.7	69, 24.2	
Pre-war lifestyle characteristics (n,%)					
Healthy diet^c^	831, 83.4	455, 81.0	123, 82.0	253, 88.6	0.014^a^ ^,^ ^b^
Regular physical activity (PA)^d^	444, 46.4	251, 46.6	71, 50.4	122, 44.2	0.490
Non-smoking^e^	771, 77.3	415, 73.8	103, 68.7	253, 88.8	<0.001^a^ ^,^ ^b^
Normal weight	476, 53.5	255, 51.2	75, 56.4	146, 56.6	0.134
Pre-war healthy lifestyle^f^	535, 53.6	282, 50.2	77, 51.3	175, 61.4	0.007

^a^ A significant difference between participants displaced in hotels and non-displaced participants.

^b^ A significant difference between participants displaced in apartments and non-displaced participants.

^c^ A healthy diet was defined as a diet quality score of ≥5.

^d^ Regular PA, was defined as PA frequency of ≥3 times per week.

^e^ Non-smoking was defined as never smoking or former smoking.

^f^ A pre-war healthy lifestyle was determined when ≥3 healthy lifestyle characteristics were reported.

### Immediate Changes in Lifestyle and Body Weight During the War

The majority of the participants rated their pre-war diet as high quality (83.4%). PA was performed regularly (≥once/week) by 78.9% of the survey population before the war, while 46.4% reported performing PA ≥3 times a week. Only 22.7% reported smoking regularly before the war or in the past, and the rest had never smoked. Fifty-three percent of the survey population described their body weight as normal, 5.1% as underweight and 41.4% as overweight. Overall, the pre-war healthy lifestyle distribution among the survey population was: low 17.2%, medium 29.3%, high 34.1%, and very high 19.5%.

**FIGURE 1:**
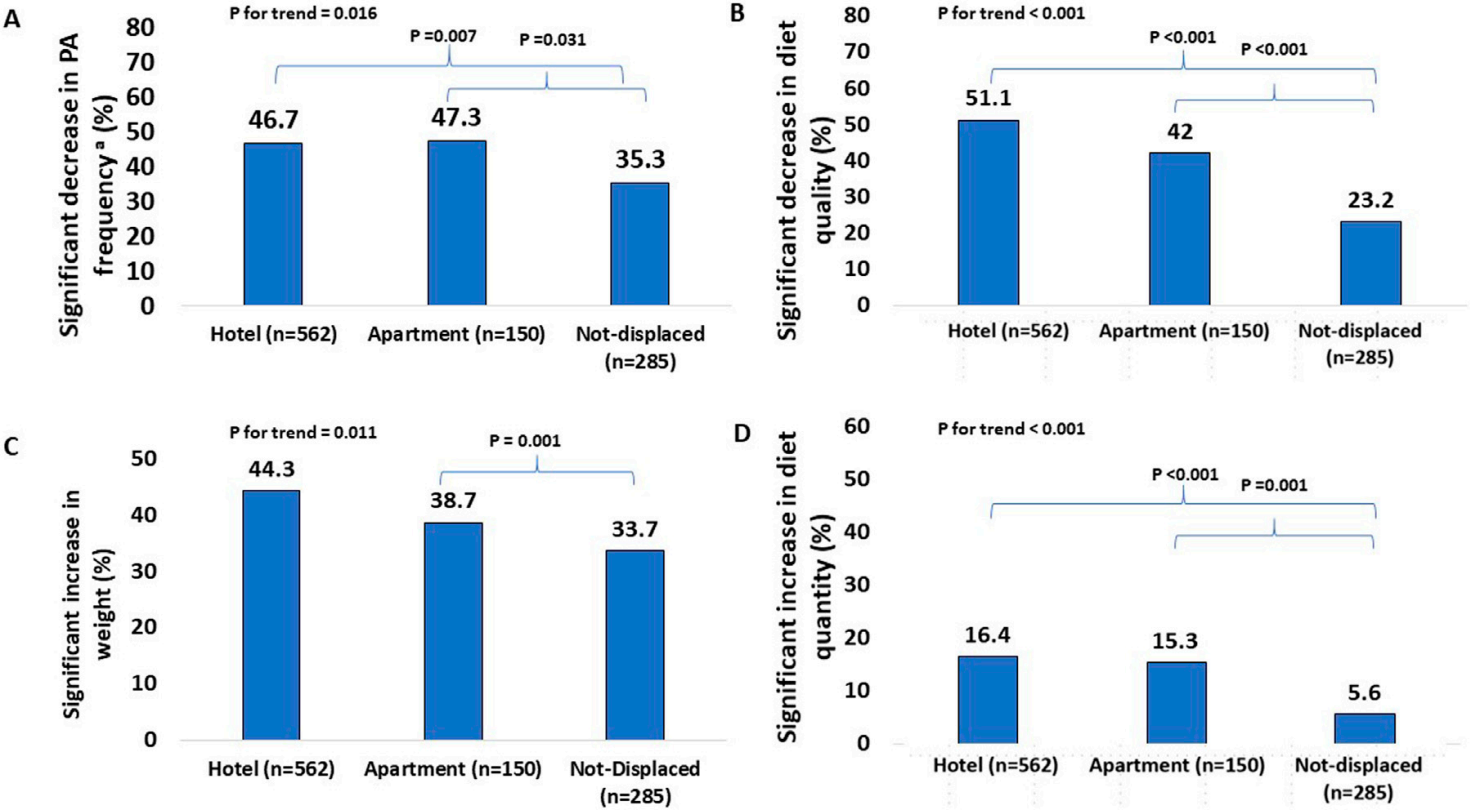
Comparison of changes in lifestyle characteristics between groups, Israel, 2024. ^a^A decrease in PA frequency among those who performed PA regularly before the war.

During the war, 75.0% of chronic smokers reported an increase in their smoking burden. Interestingly, another 3.9% (n = 37) stated that they did not smoke before the war but started smoking during the war. Among participants who reported pre-war PA levels (≥1 time per week), 43.5% reported a significant decrease in PA frequency. Forty-two percent of the survey population reported a significant decrease in their diet quality, and 13.1% reported a significant increase in diet quantity during the war. Weight gain during the war was reported by 40.4% (n = 403) of the survey population, with 10.4% reporting a significant increase in weight (n = 104). Interestingly, 15.9% (n = 159) of participants reported weight loss during the war, and 3.1% (n = 31) reported significant weight loss. The reported change in body weight was significantly correlated with changes in diet quality (P < 0.001), diet quantity (P < 0.001), PA frequency (P < 0.001) and smoking (P = 0.008). A decrease in diet quality, and PA frequency, and an increase in diet quantity and body weight, were significantly associated with place of stay during the war (Figure 1).

Displaced participants reported a higher frequency of consumption of sugar-sweetened beverages, fast foods, and red meat, and a lower frequency of consumption of whole grains and legumes (Table 2).

**TABLE 2:** Dietary characteristics of the survey population, and between group caparison, Israel, 2024.

	Total population (N = 997)	Displaced in hotels (n = 562)	Displaced in apartments (n = 150)	Not displaced (n = 285)	P P For trend
Harmful food groups n, (% of respondents)					
Sugar-sweetened beverages (n = 750) (≥3 per week)	164, 21.9	113, 27.1	25, 22.1	26, 11.8	<0.001^a^ ^,^ ^b^ <0.001
Fast food (n = 796) (≥3 per week)	256, 32.2	188, 42.0	36, 31.0	32, 13.8	<0.001^a^ ^,^ ^b^ <0.001
Red meat (n = 809) (≥3 per week)	162, 20.0	103, 22.8	24, 19.7	35, 14.9	0.049^a^ 0.044
Savory snacks (n = 688) (≥3 per week)	197, 28.6	110, 28.1	37, 38.9	50, 24.8	0.039^b^ 0.044
Sweets and desserts (n = 681) (≥3 per week)	471, 69.2	249, 66.8	80, 76.2	142, 70.0	0.173 0.164
Beneficial food groups n, (% of respondents)					
Whole grains (n = 724) (≥3 per week)	206, 28.5	96, 22.9	26, 25.2	84, 41.8	<0.001^a^ ^,^ ^b^ <0.001
Legumes (n = 687) (≥3 per week)	151, 22.0	68, 17.0	24, 24.0	59, 31.4	<0.001^a^ 0.001
Vegetables (n = 696) (>2 per day)	393, 56.5	218, 55.2	70, 68.6	105, 52.8	0.023^b^ 0.021
Fruits (n = 693) (>2 per day)	574, 82.8	335, 88.4	83, 80.6	156, 73.9	<0.001^a^ <0.001

^a^ A significant difference between participants displaced in hotels and non-displaced participants.

^b^ A significant difference between participants displaced in apartments and non-displaced participants.

P for trend indicates the trend of the proportions between non-displaced, displaced in apartments, and displaced in hotels.

**FIGURE 2:**
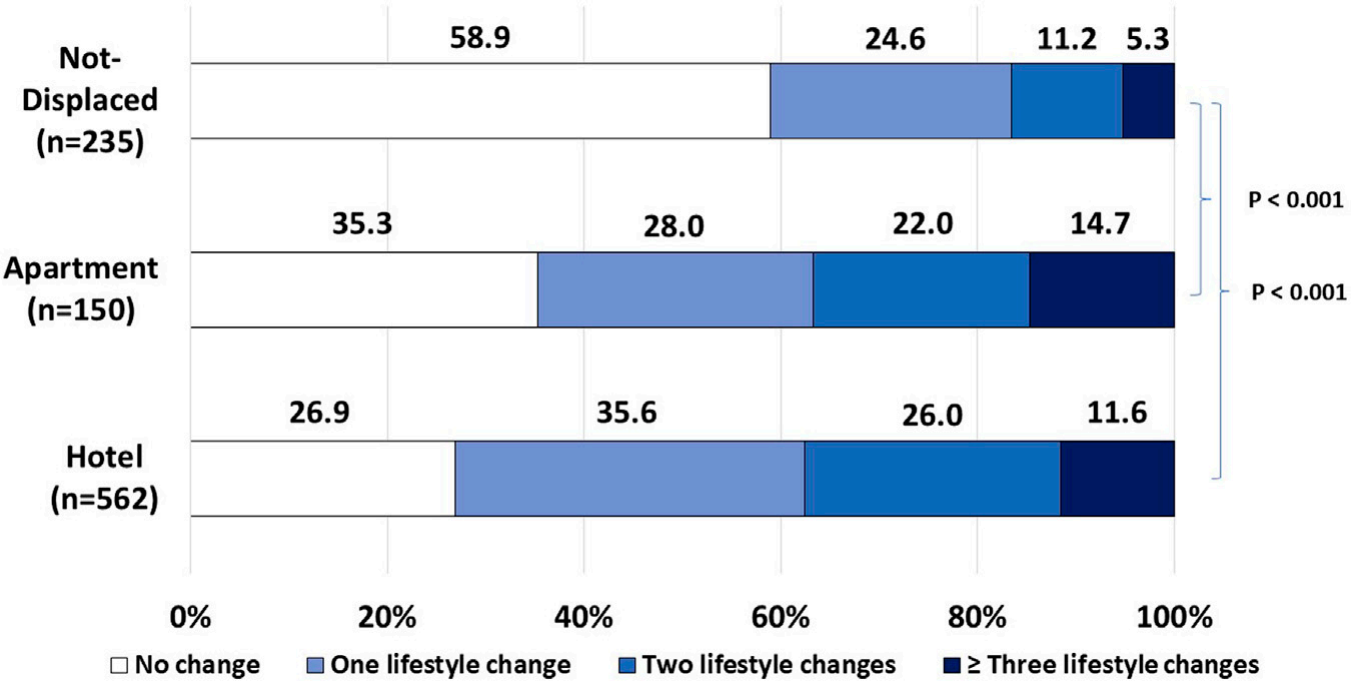
Number of unhealthy behavioral lifestyle changes between groups, Israel, 2024. Legend: Lifestyle changes include a significant decrease in diet quality, PA frequency, and an increase in diet quantity, smoking burden and alcohol consumption.

Overall, a significant negative association between displacement and the number of lifestyle changes was observed. While non-displaced participants reported higher rates of unchanged lifestyle behaviors, displaced participants reported significantly higher rates of multiple unhealthy lifestyle changes (Figure 2).

**FIGURE 3:**
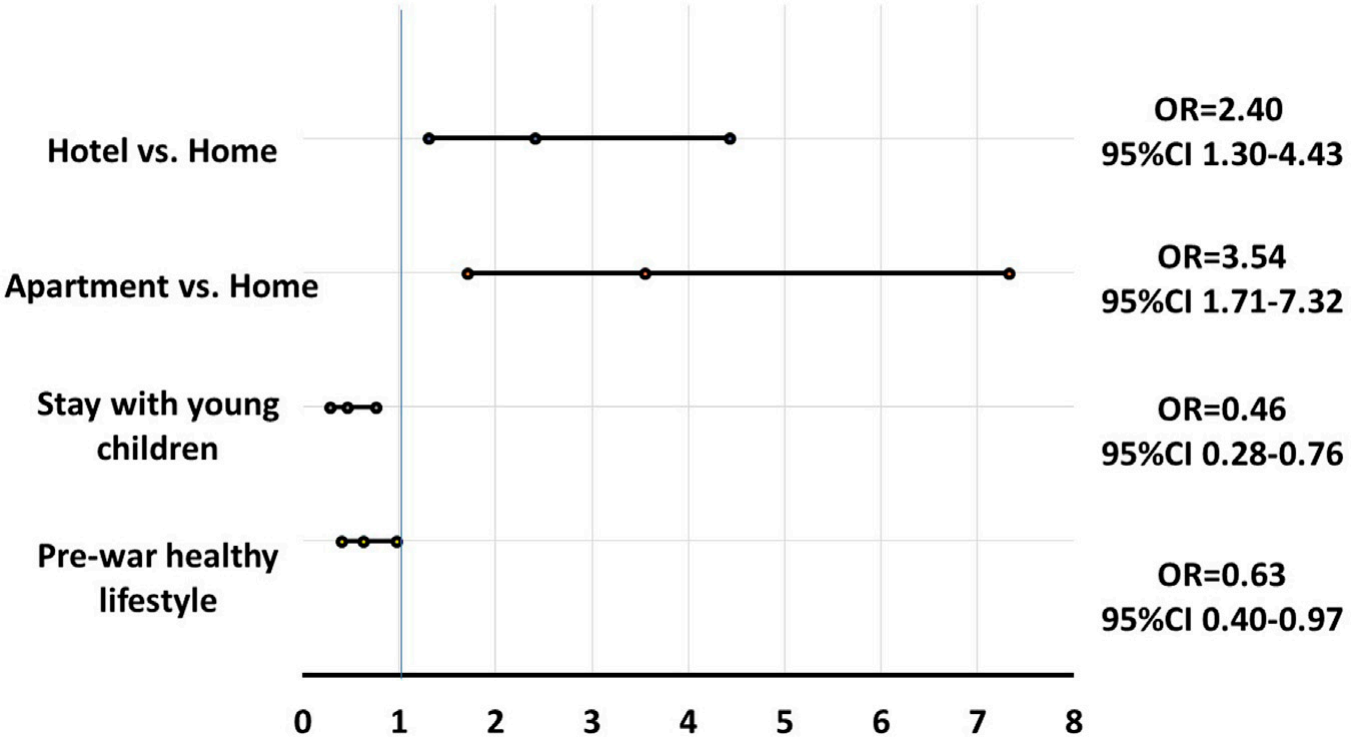
Multivariate analysis for detrimental lifestyle changes during the war (N = 980), Israel, 2024. Legend: ORs are adjusted for age, gender, chronic disease, and religion, and to one another.

In multivariate analysis, living with young children, and a pre-war healthy lifestyle were independently protective against detrimental lifestyle changes (OR = 0.46, 95%CI 0.28–0.76 and OR = 0.63, 95%CI 0.40–0.97 respectively), while staying in a hotel, or an apartment were risk factors (OR = 2.40, 95%CI 1.30–4.43 and OR = 3.54, 95% CI 1.71–7.32 respectively) (Figure 3).

### Late-Term Changes in Lifestyle and Body Weight During the War

In the second survey, 8-9 months after displacement 154 participants (age 19–93 years, 76.6% women), responded to the questionnaire. Fifty-eight percent were living in hotels, 19% in apartments and 23% at home. Months into the war, the vast majority of participants reported negative changes in their lifestyle as a result of the conflict. Compared to before the war, 55.2% of respondents reported a significant decrease in the frequency of PA, 38.3% reported a significant decrease in the quality of their diet, and 20.8% reported a significant increase in their weight. Smaller proportions of participants reported increases in smoking frequency (11.6%) and in alcohol consumption (13.6%). Among participants displaced in hotels, 71% reported a dramatic increase in their body weight (compared to 9.4% of those displaced in apartments and 18.8% of those at home (P = 0.033)). In addition 77.6% of the respondents reported a high intake of processed meat (compared to 12.2% of those displaced in apartments and 10.2% of those at home, P < 0.001), and 39.4% reported a high intake of fast food (compared to 17.9% of those displaced in apartments and 5.7% of those at home, P < 0.001). The reported intake of other health-related lifestyle habits did not differ between groups.

## Discussion

This study examined the health risks associated with population displacement during times of crisis using a population-based survey. Population displacement is a commonly used measure to protect civilians during times of conflict, natural disasters or climatic disasters and economic crisis [16, 17]. In 2022, the number of displaced people worldwide reached 100 million [18]. Forced displacement can have major effects on the mental and physical health of populations in both developing [4, 19, 20] and developed countries [9, 13, 21]. Our survey population provided a unique perspective on the potential health effects of internal displacement of a largely healthy population in a developed country shortly after displacement. However, although relatively high rates of adherence to health-promoting activities were reported in our survey population before the war, harmful dietary and lifestyle changes were common during the war immediately after displacement. These were stable among those displaced in hotels, throughout the months after displacement as seen in our second survey, validating the short-term results found relatively close to displacement. Forcibly displaced participants, whether in hotels or apartments, reported a decrease in diet quality and PA frequency, and an increase in smoking burden, compared to those who were not displaced. The reported change in diet quality and quantity was consistent with increased consumption of fast food, red meat and sugar-sweetened beverages. This change in diet may have been predictable among participants displaced in hotels, due to the lack of control over elementary food choices (such as what to eat, how to prepare food and when to eat), although the results from displaced populations in apartments were similar. This may imply that the lack of access and control over one’s food preparation and the eating environment in hotels are not the only detrimental contributors to these reported outcomes. Concerns may be raised regarding the mental capabilities of the forcibly displaced, which limited their ability to control this aspect of their day-to-day lives, and was common to both those displaced in hotels and those displaced in apartments. Interestingly, in our follow-up survey, the negative health effects of displacement were reported only by those in hotels, while those in apartments appeared to have retained some of their previous lifestyle. This result may imply that over time, certain behavioral adaptations were more prevalent among those displaced in apartments, potentially due to their control over basic food choices. In contrast, those displaced in hotels could not adapt due to their living circumstances, and continued to suffer from the detrimental effects of displacement on health-promoting lifestyle.

There are several potential explanations for our findings. First, as has been previously shown, displacement may have extreme mental effects [6, 8, 22], due to the detachment from normal daily routine activities such as livelihoods or education, family and community, and in some cases due to traumatic personal or family experiences [7, 23]. Second, displacement often increases financial hardship [19, 24], access to education and social mobility [25]. For these and other reasons, the displaced may have limited mental focus, priority, or motivation, and face financial inability to maintain a healthy lifestyle.

Finally, immediate weight gain was reported by 40.4% of the survey population, with significantly higher rates among participants displaced in hotels compared to those who were not displaced. A similar result was also seen 8-9 months after displacement, suggesting that detrimental changes to health promoting lifestyle habits are especially difficult to maintain in hotels, even after months of potential adjustment. Weight gain is an established risk factor for many chronic diseases, and obesity is at the core of public health goals worldwide [26, 27]. It is especially undesirable in individuals with a background of metabolic disease [28, 29]. Our survey population included 18% of respondents with reported chronic disease, who are specifically instructed to maintain a healthy lifestyle and body weight as part of their disease management [30]. Therefore, this subpopulation is at a particularly higher risk for disease progression. These findings are consistent with previous studies that have associated long-term displacement during times of war with an increased risk of obesity-related diseases [4, 31]. This study, which focused on the short-term events following displacement, was able to shed light on the initiation of risk accumulation for chronic diseases. As displacement resulting from crises around the world and in Israel can be long-term, the results of this study may point to an ongoing threat to the health of a large population in Israel.

In multivariate analysis, displacement in hotels or in apartments was a risk factor for detrimental lifestyle changes during the war. Interestingly, living with young children was a protective factor, a result that may reflect the potential of some family eating habits to encourage a healthy lifestyle. These may include family meals eaten together, parents’ efforts to set good examples for their children, and the need to maintain a daily routine with fixed meal times [32, 33]. Another interesting protective factor against detrimental lifestyle changes was a pre-war healthy lifestyle, implying the importance of health awareness, continuity of habits and stability in times of crisis.

It is also important to mention that 15.9% of our survey population reported significant weight loss, potentially reflecting loss of appetite and reduced food intake under the stressful conditions of war [34].

This study emphasizes the need for public health interventions when managing a crisis-induced forced internal displacement of citizens for long periods of time in a developed country. While the safety of the population is of the utmost importance, the displaced have many fundamental health needs, which include a healthy diet and the availability of opportunities to exercise in order to maintain a healthy weight. Over time, public health dieticians in Israel have developed community interventions in hotels to facilitate the availability of these measures, changes in hotel menus and portions served, and raising awareness through public campaigns.

The strengths of this study include a relatively large sample size of forcibly displaced participants, the detailed information collected from them, and both a relatively early assessment of the short-term effects of displacement and a late assessment months later. It is possible that even participants who did not report weight gain in our survey, but who experienced detrimental lifestyle changes, may develop weight gain in the near future. Early identification of risk factors for the development and progression of chronic diseases is the basis of primary prevention, so that they can be removed [35, 36]. Thus, the results of this study identify the forcibly displaced as a vulnerable population and provide an opportunity for prevention measures.

Limitations of this study include the sampling frame and method, which limit the external validity of the study population, especially in the case of non-displaced participants. Due to its observational nature, this survey is subject to information bias: data were reported by participants and were not objectively measured, so reporting and recall bias may be present, and our results should be interpreted as merely representing a behavioral trend. The questionnaire did not include questions that could provide a deeper understanding as to the effect of the war on participants, such as information about endurance of the various forms of violence during the terror attacks of 7 October, loss or physical injury of family members and close friends, damage to family property and livelihood, or having family members actively participating in the battles. We also deliberately did not include questions on pre-war mental conditions, which may have been detrimental to the coping capacity of individuals and households. Other important health-related outcomes that were not assessed include adherence to medication treatment, attendance at clinic visits or completion of screening tests. Finally, we did not account for the distance between the original place of residence and the place of displacement, which could enhance our understanding of the effect of differences in geographic location and living conditions on the reaction to displacement.

### Conclusions

Forcibly displaced populations, staying in hotels, may be at increased risk of developing obesity and related chronic diseases, due to detrimental changes in health promoting behaviors, particularly diet and PA. Future studies should aim to assess the long-term lifestyle-related health consequences of displacement in developed countries. Tailored preventive measures should be developed and implemented, to reduce the potential additional health burden inflicted on this population.

## Data Availability

The datasets used and/or analyzed in the current study are available from the corresponding author upon reasonable request.

## References

[1] Loretti A. Armed Conflicts, Health and Health Services in Africa. An Epidemiological Framework of Reference. Med Confl Surviv (1997) 13(3):219–28. 10.1080/13623699708409342 9290329

[2] Savona-Ventura C Mahmood T Mukhopadhyay S Martins N Louwen F Tarlatzis B. The Consequences of Armed Conflict on the Health of Women and Newborn and Sexual Reproductive Health - A Position Statement by the European Board and College of Obstetrics and Gynaecology (EBCOG). Eur J Obstet Gynecol Reprod Biol (2022) 274:80–2. 10.1016/j.ejogrb.2022.05.015 35609350

[3] Haftu H Weledegebriel MG Gebre-Egziabher A Gebrehiwot T Zenebe D Berhe B Experience Sharing on Continuity of Healthcare Services in Internally Displaced Peoples: The Case of Tigray War Crisis. Risk Manag Healthc Policy (2023) 16:2197–208. 10.2147/RMHP.S426627 37881166 PMC10595205

[4] Doocy S Sirois A Tileva M Storey JD Burnham G. Chronic Disease and Disability Among Iraqi Populations Displaced in Jordan and Syria. Int J Health Plann Manage (2013) 28(1):e1–12. 10.1002/hpm.2119 22685057

[5] Calam R. Public Health Implications and Risks for Children and Families Resettled after Exposure to Armed Conflict and Displacement. Scand J Public Health (2017) 45(3):209–11. 10.1177/1403494816675776 27799420

[6] Zabłocka-Żytka L Lavdas M. The Stress of War. Recommendations for the Protection of Mental Health and Wellbeing for Both Ukrainian Refugees as Well as Poles Supporting Them. Psychiatr Pol (2023) 57(4):729–46. 10.12740/PP/156157 38170647

[7] Bunn M Khanna D Farmer E Esbrook E Ellis H Richard A Rethinking Mental Healthcare for Refugees. SSM Ment Health (2023) 3:100196. 10.1016/j.ssmmh.2023.100196 37501680 PMC10370474

[8] Boiko DI Shyrai PO Mats OV Karpik ZI Rahman MH Khan AA Mental Health and Sleep Disturbances Among Ukrainian Refugees in the Context of Russian-Ukrainian War: A Preliminary Result from Online-Survey. Sleep Med (2024) 113:342–8. 10.1016/j.sleep.2023.12.004 38104463

[9] Lushchak O Velykodna M Bolman S Strilbytska O Berezovskyi V Storey KB. Prevalence of Stress, Anxiety, and Symptoms of Post-traumatic Stress Disorder Among Ukrainians after the First Year of Russian Invasion: A Nationwide Cross-Sectional Study. Lancet Reg Health Eur (2024) 36:100773. 10.1016/j.lanepe.2023.100773 38019977 PMC10665943

[10] Tambe AB Akeh ML Tendongfor N Dhlamini T Chipili G Mbhenyane X. The Predictors of Food Security and Dietary Diversity Among Internally Displaced Persons’ Children (6-59 Months) in Bamenda Health District, Cameroon. Confl Health (2023) 17(1):11. 10.1186/s13031-023-00511-2 36959669 PMC10035966

[11] Nisbet C Lestrat KE Vatanparast H. Food Security Interventions Among Refugees Around the Globe: A Scoping Review. Nutrients (2022) 14(3):522. 10.3390/nu14030522 35276878 PMC8839314

[12] Bernal O Garcia-Betancourt T León-Giraldo S Rodríguez LM González-Uribe C. Impact of the Armed Conflict in Colombia: Consequences in the Health System, Response and Challenges. Confl Health (2024) 18(1):4. 10.1186/s13031-023-00561-6 38172982 PMC10762784

[13] Summers A Bilukha OO. Suboptimal Infant and Young Child Feeding Practices Among Internally Displaced Persons during Conflict in Eastern Ukraine. Public Health Nutr (2018) 21(5):917–26. 10.1017/S1368980017003421 29268805 PMC5848760

[14] Artzi-Medvedik R Tsikholska L Chertok IA. A Qualitative Exploration of the Experience of Child Feeding Among Ukrainian Refugee and Immigrant Mothers during Escape and Relocation. J Pediatr Health Care Off Publ Natl Assoc Pediatr Nurse Assoc Pract (2024) 38(1):21–9. 10.1016/j.pedhc.2023.08.004 37747386

[15] Myronyuk IS Slabkiy GO Bilak-Lukianchuk VJ Brych VV Kondratskyi VI. The Attitude of Certain Categories of the Population of Ukraine to Personal Health during the War against Russian Aggression. Wiadomosci Lek Wars Pol (1960) 76(8):1883–7. 10.36740/wlek202308126 37768787

[16] Climate Change and Population Displacement: Disasters and Diasporas in. (2024) Available online at: https://www.taylorfrancis.com/chapters/edit/10.4324/9781315434773-6/climate-change-population-displacement-disasters-diasporas-twenty-first-century-anthony-oliver-smith (Accessed: February 2, 2024).

[17] Bellizzi S Popescu C Panu Napodano CM Fiamma M Cegolon L. Global Health, Climate Change and Migration: The Need for Recognition of “Climate refugees.”. J Glob Health (2025) 13:03011. 10.7189/jogh.13.03011 PMC10037158 36960688

[18] United Nations Office for the Coordination of Humanitarian Affairs 2023. Global Humanitarian Overview. (2024) 184. 10.18356/9789210024136

[19] Kaiser BN Ticao C Boglosa J Minto J Chikwiramadara C Tucker M Mental Health and Psychosocial Support Needs Among People Displaced by Boko Haram in Nigeria. Glob Public Health (2020) 15(3):358–71. 10.1080/17441692.2019.1665082 31535595 PMC7028461

[20] Arage MW Kumsa H Asfaw MS Kassaw AT Dagnew EM Tunta A Exploring the Health Consequences of Armed Conflict: The Perspective of Northeast Ethiopia, 2022: A Qualitative Study. BMC Public Health (2023) 23(1):2078. 10.1186/s12889-023-16983-z 37875885 PMC10594710

[21] Badanta B Márquez De la Plata-Blasco M Lucchetti G González-Cano-Caballero M. The Social and Health Consequences of the War for Ukrainian Children and Adolescents: A Rapid Systematic Review. Public Health (2024) 226:74–9. 10.1016/j.puhe.2023.10.044 38007844

[22] Dissanayake L Jabir S Shepherd T Helliwell T Selvaratnam L Jayaweera K The Aftermath of War; Mental Health, Substance Use and Their Correlates with Social Support and Resilience Among Adolescents in a Post-conflict Region of Sri Lanka. Child Adolesc Psychiatry Ment Health (2023) 17(1):101. 10.1186/s13034-023-00648-1 37653394 PMC10472617

[23] Jolof L Rocca P Carlsson T. Support Interventions to Promote Health and Wellbeing Among Women With Health-Related Consequences Following Traumatic Experiences Linked to Armed Conflicts and Forced Migration: A Scoping Review. Arch Public Health Arch Belg Sante Publique (2024) 82(1):8. 10.1186/s13690-023-01235-8 PMC10790529 38225672

[24] Behrendt M Vervliet M Rota M Adeyinka S Uzureau O Rasmussen A A Conceptual Study on the Relationship between Daily Stressors, Stressful Life Events, and Mental Health in Refugees Using Network Analysis. Front Psychol (2023) 14:1134667. 10.3389/fpsyg.2023.1134667 37599778 PMC10438848

[25] The Ripple Effect: Economic Impacts of Internal Displacement. Koninklijke Brill NV Geneva, Switzerland: Internal Displacement Monitoring Center (IDMC). (2024) Available online at: https://primarysources.brillonline.com/browse/human-rights-documents-online/the-ripple-effect-economic-impacts-of-internal-displacement;hrdhrd9806201898060010 (Accessed: February 2, 2024).

[26] James WPT. WHO Recognition of the Global Obesity Epidemic. Int J Obes (2008) 32(Suppl. 7):S120–6. 10.1038/ijo.2008.247 19136980

[27] Twig G Reichman B Afek A Derazne E Hamiel U Furer A Severe Obesity and Cardio-Metabolic Comorbidities: A Nationwide Study of 2.8 Million Adolescents. Int J Obes (2019) 43(7):1391–9. 10.1038/s41366-018-0213-z 30258119

[28] Wolfenden L Ezzati M Larijani B Dietz W. The Challenge for Global Health Systems in Preventing and Managing Obesity. Obes Rev Off J Int Assoc Study Obes (2019) 20(Suppl. 2):185–93. 10.1111/obr.12872 31317659

[29] Dietz WH Baur LA Hall K Puhl RM Taveras EM Uauy R Management of Obesity: Improvement of Health-Care Training and Systems for Prevention and Care. Lancet Lond Engl (2015) 385(9986):2521–33. 10.1016/S0140-6736(14)61748-7 25703112

[30] de Faria RR de Siqueira SF Haddad FA Del Monte Silva G Spaggiari CV Martinelli Filho M. The Six Pillars of Lifestyle Medicine in Managing Noncommunicable Diseases - the Gaps in Current Guidelines. Arq Bras Cardiol (2024) 120(12):e20230408. 10.36660/abc.20230408 38198361 PMC10735241

[31] Yazbeck N Mansour R Salame H Chahine NB Hoteit M. The Ukraine-Russia War Is Deepening Food Insecurity, Unhealthy Dietary Patterns and the Lack of Dietary Diversity in Lebanon: Prevalence, Correlates and Findings from a National Cross-Sectional Study. Nutrients (2022) 14(17):3504. 10.3390/nu14173504 36079761 PMC9460330

[32] Snuggs S Harvey K. Family Mealtimes: A Systematic Umbrella Review of Characteristics, Correlates, Outcomes and Interventions. Nutrients (2023) 15(13):2841. 10.3390/nu15132841 37447168 PMC10346164

[33] Dos Santos CT Machado CO Hofelmann DA. Family Meals, Diet Quality and Obesity Among Adolescents: Findings from a Schoolbased Study in a Capital City of Southern Brazil. Minerva Pediatr (2021) 76. 10.23736/s2724-5276.20.05918-6 33820405

[34] Wallis DJ Hetherington MM. Emotions and Eating. Self-Reported and Experimentally Induced Changes in Food Intake under Stress. Appetite (2009) 52(2):355–62. 10.1016/j.appet.2008.11.007 19071171

[35] Pearce C Rychetnik L Wutzke S Wilson A. Obesity Prevention and the Role of Hospital and Community-Based Health Services: A Scoping Review. BMC Health Serv Res (2019) 19:453. 10.1186/s12913-019-4262-3 31277640 PMC6612151

[36] Pearce C Rychetnik L Wilson A. The Obesity Paradigm and the Role of Health Services in Obesity Prevention: A Grounded Theory Approach. BMC Health Serv Res (2021) 21:111. 10.1186/s12913-021-06089-w 33526017 PMC7851945

