# Supplementary material for: Detrimental Changes in Individual Health-Promoting Behaviors Among Internally Displaced Israelis
Source: Int J Public Health. 2025 May 21;70:1607794. doi: 10.3389/ijph.2025.1607794 (PMC12133527; doi:10.3389/ijph.2025.1607794)
Supplement: Supplementary file 1 [file DataSheet1.pdf]

Supplementary material

Supplementary table 1. Comparison between participants who fully or partially answered the questionnaire, Israel, 2024

	Fully answered questionnaire (n=993)	Partially answered questionnaire (n=68)[a]	P
Age (mean±sd)	47.5±16.4	41.1±15.8	0.001
Gender (% female)	827, 82.9%	56, 82.3%	0.889
Reported chronic disease (n,%)	159, 16.9%	9, 2.1%	<0.001
Not Displaced (n,%)	369, 37.0%	26, 6.6%	<0.001
Stay with children (under 18y) (n,%)	444, 45.1%	24, 36.4%	0.168

[a] Information regarding demographics was available for a sub-sample of participants who did not fully answer the questionnaire

Supplementary file 1. Study questionnaire

SURVEY OF DIETARY HABITS DURING THE WAR

In these times, many of us have experienced significant changes to our eating habits and lifestyles.

Thank you for your consent to fill out this questionnaire regarding your food habits.

The answers you give will enable us to know more about the eating habits of the Israeli population during a time of war, as to identify nutritional needs.

All of your answers will be confidential, and will only serve the nutrition staff and research teams in your local authority, together with the Nutrition Division of the Ministry of Health.

There are no wrong answers! Please answer as honestly as possible.

The completion time is about 5-10 minutes.

Thank you very much for your cooperation.

Answering the questions is voluntary and not mandatory. Answering the questionnaire is taken as your consent to take part in the survey. In answering the survey, I agree that the data provided by me will serve the interviewers for internal purposes only.

☐ I agree to answer the questionnaire, as my answer are used by the investigators of the Ministry of Health

Demographic details

We thank you if you could answer a few background questions. The questionnaire is anonymous, and your details remain confidential.

1. What is your year of birth? ____________________

2. What is your gender?

a. Male
b. Female
c. Other

3. What is the name of your permanent town/city of residence?

---

4. Where are you currently residing?

a. Hotel / Absorption facility
b. An apartment (paid, of family or friends)
c. At home
d. Other, specify: ______________________________

5. In which city/town is the hotel/absorption facility? ________________________________

6. Are your children/children under the age of 18 living with you?

a. Yes
b. No

7. Are you a:

a. Jew
b. Moslem
c. Moslem Bedouin
d. Christian Arab
e. Christian
f. Druze
g. Other, specify: _________________________

8. Do you regard yourself as:

a. Secular
b. Religious / traditional
c. Ultraorthodox
d. Other ______________________________________

9. Have you been diagnosed with a medical condition which limits your food intake? Have you received medical instructions to change your eating habits?

a. Yes (go to 10a)
b. No

9a. Please give details of your medical condition__________________________________

The following questions refer to your lifestyle before, and during the war

1. How would you estimate the quality of your diet, how healthy is your dietary intake, **usually i.e. before the war**? (circle your answer)

0 (not healthy at all) 1 2 3 4 5 6 7 8 9 10 (very healthy eating habits)

2. Has there been a change in the quality of your diet, how healthy is your dietary intake, now as compared to before the war?

a. I estimate that the quality of my diet is similar to that before the war
b. I estimate that the quality of my diet has slightly decreased compared to that before the war
c. I estimate that the quality of my diet has significantly decreased compared to that before the war
d. I estimate that the quality of my diet has slightly improved compared to that before the war
e. I estimate that the quality of my diet has significantly improved compared to that before the war

3. Has there been a change in the amount of food you eat **now, compared to before the war?**

a. I estimate that I eat a similar amount compared to what I ate before the war
b. I estimate that I eat slightly less compared to what I ate before the war
c. I estimate theta I eat significantly less compared to what I ate before the war
d. I estimate that I eat slightly more compared to what I ate before the war
e. I estimate that I eat significantly more compared to what I ate before the war

4. How do you assess the quality of your children's diet? How healthy was it? Before the war? (circle your answer)

0 (not healthy at all) 1 2 3 4 5 6 7 8 9 10 (very healthy eating habits)

5. Has there been a change in your children's dietary quality now, as compared to before the war?

a. I estimate that the quality of their diets is similar to what was before the war
b. I estimate that the quality of my children's diets has slightly decreased during the war

c. I estimate that the quality of my children's diets has <u>significantly decreased</u> during the war
d. I estimate that the quality of my children's diets has <u>slightly improved</u> during the war
e. I estimate that the quality of my children's diets has <u>significantly improved</u> during the war

6. On average, how many times a week did you exercise before the war (between 0-7 sessions a week)

0 (don't do any exercise) 1 2 3 4 5 6 7 (sessions a week)

7. Has there been a change in your frequency of physical activity, **<u>now, as compared to before the war?</u>**

a. I estimate that my frequency of physical activity is the <u>same</u> now as it was before the war
b. I estimate that my frequency of physical activity has <u>slightly decreased</u> during the war
c. I estimate that my frequency of physical activity has <u>significantly decreased</u> during the war
d. I estimate that my frequency of physical activity has <u>slightly improved</u> during the war
e. I estimate that my frequency of physical activity has <u>significantly improved</u> during the war

8. Has there been a change in your <u>frequency of drinking alcohol</u> **now, as compared to usual times before the war?**

a. I estimate that my frequency of consuming alcohol is the <u>same</u> as it was before the war
b. I estimate that my frequency of consuming alcohol has <u>slightly decreased</u> compared to what as it was before the war
c. I estimate that my frequency of consuming alcohol has <u>significantly decreased</u> compared to what as it was before the war
d. I estimate that my frequency of consuming alcohol has <u>slightly increased</u> compared to what as it was before the war
e. I estimate that my frequency of consuming alcohol has <u>significantly increased</u> compared to what as it was before the war
f. I don't drink alcohol at all

9. Do you smoke cigarettes/tobacco?

a. I have never smoked
b. I don’t smoke now but I did in the past
c. I smoke now (go to 9a)
d. I didn't usually smoke before the war but lately I have started to smoke

9a. Has there has been a change in the frequency of your smoking **now, as compared to usual times before the war?**

a. I smoked before the war and I smoke now at the same frequency
b. Before the war, I smoked less frequently
c. Before the war, I smoked more frequently

10. How do you assess your weight, compared to a normal weight to your age and gender group, **in usual times, before the war**?

a. Less than desirable
b. Normal, average for my age
c. Greater than desirable

11. Has there been a **change in your weight now, as compared to the usual state, before the war?**

a. I estimate that my weight now is the same as it was before the war
b. I assume that my weight has slightly decreased during the war
c. I estimate that my weight has significantly decreased during the war
d. I estimate that my weight has slightly increased during the war
e. I estimate that my weight has significantly increased during the war

Thank for your answers up to this point. The following questions relate to your current eating habits, since the outbreak of war.

1. Below is a table with a list of food groups. For each group, please indicate the average frequency of consumption. Please indicate the frequency of consumption of the following food groups now, since the outbreak of war.

(Only one answer per line permitted)

	Food group	Less than once a week/ never	1-2 a week	3-4 a week	5-6 a week	1 daily	2-3 daily	4-5 daily	6 or more daily
a	**Vegetables** fresh, cooked, baked and other								
b	**Fruit-fresh** not including juice, dried, or canned fruit								
c	**Legumes** lentils, white beans, chickpeas, including homemade/home style - humus spread, peas, mung beans, tofu								
d	**<u>Whole</u> grains** whole wheat bread, wholegrain pasta, bulgur, frika, buckwheat, barley, whole rice, oats								
e	**Desserts and sweets-** cakes, cookies, wafers, chocolate, ice-cream, confectionery								
f	**Savory/salty snacks**								
g	**Savory baked goods**								
h	**Sugar-sweetened drinks** soft drinks/sodas, fruit juice, energy drinks,								

	cola, carbonated drinks, ice tea, iced drinks								
i	**Processed meat products**, **prepared meats** sausage, hamburger, kebab, ready frozen shnitzel, smoked meats and fish								
j	**Fresh meat (not processed)** such as meals prepared from fresh beef/lamb/veal								
k	**Sauces/dressings** ketchup, mayonnaise, sweet chili sauce, teriyaki, 1000 Island dressing and others								
l	**Fast foods or fried foods**- French fries, onion rings, pizza and others								

2. We are interested to hear your further thoughts regarding your eating habits or those of your family, now, since the outbreak of war.

Thank you very much!

We value the time and thought you have given to completion of the questionnaire!

We wish you and your family good health, and we hope for better and calmer days soon!

Study team

Nutrition division

Ministry of health
